# *π*-Electron ferromagnetism of a purely organic radical crystal

**Published:** 2004-02-01

**Authors:** Minoru Kinoshita

**Affiliations:** Professor Emeritus, University of Tokyo

**Keywords:** Organic ferromagnet, organic radical, galvinoxyl, *p*-nitrophenyl nitronyl nitroxide (*p*-NPNN), ferromagnetic interaction, spin polarization

## Abstract

Transition to ferromagnetic order was found in the early 1990’s with a crystal of a purely organic compound without any metallic elements. The process of the research is reviewed starting from the pre-stage study to obtain the designing strategies to bring out ferromagnetic interaction in organic crystal and ending at the discovery of the ferromagnetism in the crystal of *p*-nitrophenyl nitronyl nitroxide (*p*-NPNN; C_13_H_16_N_3_O_4_).

## Introduction

To endeavor at organic ferromagnets without any metal elements had long been a challenging problem in the field of physical chemistry. Researches on magnetic interaction of organic radicals had been in progress since the 1930’s keeping such interrogation in mind. In 1963, the first theoretical aspect to build up organic ferromagnets was proposed.1) Furthermore, in 1967 and 1968 as well, proposals to design them from other points of view were presented.2),3)

It was also in the 1960’s that organic compounds having intermolecular ferromagnetic (FM) interaction to align the directions of the magnetic moments in parallel were discovered experimentally. One is galvinoxyl (4- [[3,5-bis(1,1-dimethylethyl)-4-oxo-2,5,cyclohexadien-1-ylidene]methyl]-2,6-bis(1,1-dimethylethyl)phenoxy)4) and the other is TANOL-suberate.5) Likewise in the 1960’s, FM interaction was noticed within a molecule and the molecules having high spin multiplicity in a ground state were found.6),7)

A movement to search out ferromagnets with organic compounds exclusively comprised of light elements such as H, C, N and O and possessing a definite molecular weight became quite active in the 1980’s after the discovery of organic superconductors. In 1991, two successful reports for such ferromagnets became available. One of them is a crystal of a stable simple organic compound called *p*-NPNN (*p*-nitrophenyl nitronyl nitroxide)8) and the other is the one using the carbon cluster C_60_.9) In this article, our studies on *p*-NPNN are described.

## Magnetism of organic radical crystals

Almost all the organic compounds are comprised of even number of electrons, and covalent bonds are formed with two pieces of electrons in a pair. Accordingly, the magnetic moments of the electrons in each pair compensate each other resulting in diamagnetism. There are, of course, exceptional compounds called free radicals that are comprised of odd number of electrons and exhibit the magnetism caused by the spin of an unpaired electron. However, organic radicals are normally unstable or chemically reactive. To isolate them as a crystal, some contrivance is required to stabilize them. Aromatic compounds are usually planar, the ***π***-electrons are delocalized over the whole molecule, and as a result, the radicals are stabilized by electron delocalization. Another way to restrict the reactivity is chemical modifications by introducing voluminous substituent groups such as CH_3_, C(CH_3_)_3_ and C_6_H_5_, or the substituents of captodative effect.

Neutral organic radical crystals, which are usually insulators, exhibit in many cases paramagnetism in accordance with the Curie-Weiss law [*χ* = C/(T – ***Θ***)] with a negative value of ***Θ***. The negative value of ***Θ*** means that the paramagnetism becomes weaker than that expected from the Curie law due to an antiferromagnetic (AFM) interaction operating between the radicals at a low temperature. That is to say, the exchange interaction to cause the direction of the spin to be steered into antiparallel is provided even among the unpaired electrons in the neighboring radicals.

This indicates from a chemical viewpoint that a strong tendency to form covalent bond is still noted even in the radicals stabilized in the ways mentioned above, when they come close to each other in a crystal. This is a natural matter as can be seen in the formation of hydrogen molecule from two hydrogen atoms, the simplest example of radicals. Thus the AFM interaction is common, while the FM interaction is rare in organic radicals.

To design an organic ferromagnet, it is necessary to align the directions of the unpaired electron spins on the neighboring radicals in parallel. Hence the natural phenomenon where unpaired electrons are liable to form bonding should be upturned. For that purpose, several strategies previously referred to have been presented. 1),2)

**Fig. 1:**
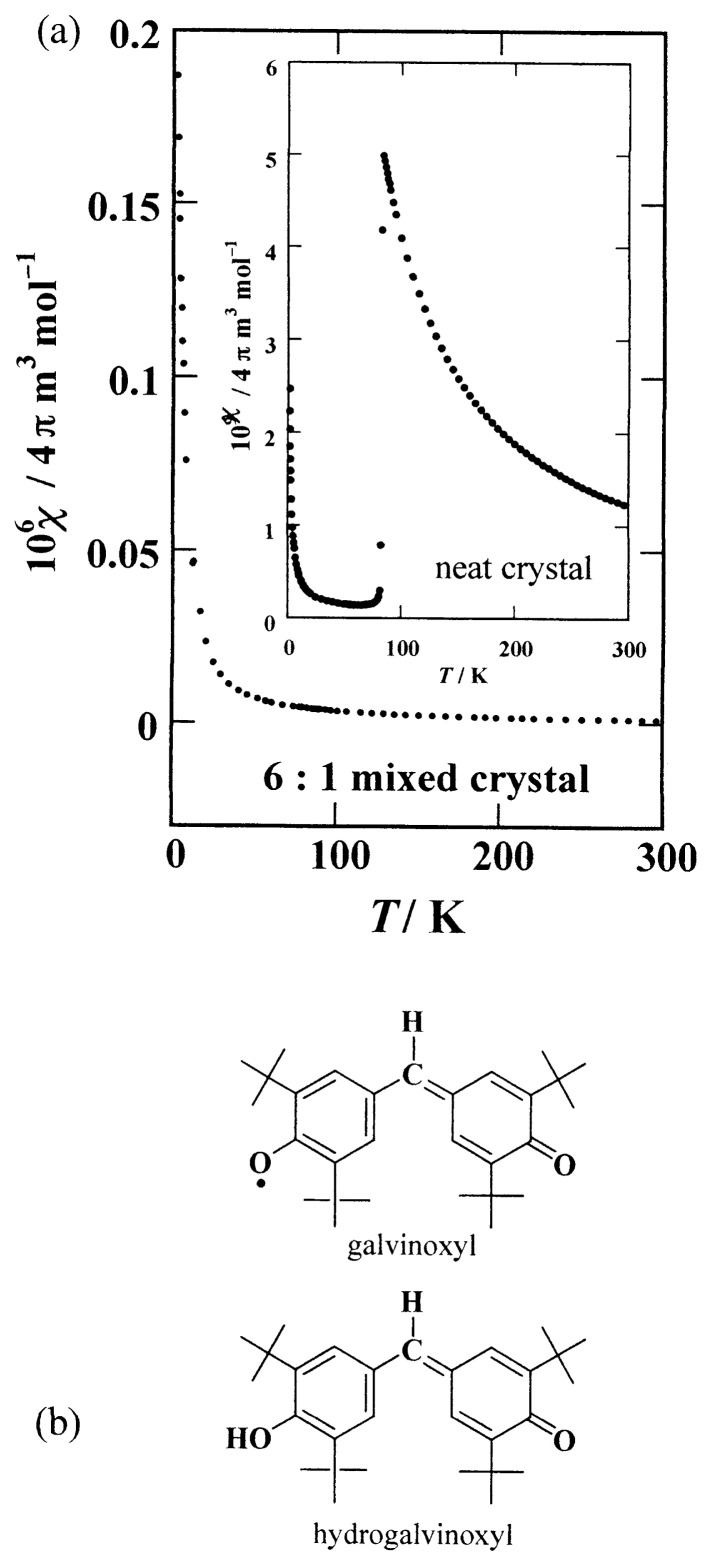
(a) Temperature dependence of the 6:1 mixed crystal of galvinoxyl and hydrogalvinoxyl. Inset shows the susceptibility of the neat galvinoxyl crystal. (b) Molecular structures of galvinoxyl and hydrogalvinoxyl.

Galvinoxyl illustrated in Fig. 1(b) was a precious example revealing that some of organic materials could have intermolecular FM coupling. Since the interaction in the galvinoxyl crystal is especially strong, it was thought that explaining its mechanism gives a crucial suggestion for designing compounds having FM interaction. We thus planned detailed studies of the galvinoxyl radical and intended to extract conditions governing its FM interaction from the study.

## Magnetism of galvinoxyl

In the inset of Fig. 1, the temperature dependence of the paramagnetic susceptibility of the galvinoxyl crystal is shown.10) The crystal undergoes a phase transition at 85 K, and becomes almost diamagnetic at the temperature below that. This had brought about difficulty in studying the interaction in the temperature region corresponding to the Weiss constant (***Θ*** = 19 K). However, we could find that the phase transition was suppressed by making mixed crystals with a small amount of hydroganoxyl (4-[[3,5-bis(1,1-dimethylethyl)-4-hydroxyphenyl]methylene] 2,6-bis(1,1-dimethylethyl)-2,5-cyclo-hexadien-1-one).10)–12) Hydrogalvinoxyl is a precursory closed shell compound of galvinoxyl, for which the crystal structure is known to be isomorphous to that of galvinoxyl, but phase change does not occur. The temperature dependence of the susceptibility of the 6:1 mixed crystal is shown in the main frame of Fig. 1. The reciprocal susceptibility of the mixed crystal crosses the temperature axis in the positive region10) (not shown), thereby confirming that the FM interaction is maintained even in the mixed crystal down to the cryogenic temperature.

**Fig. 2:**
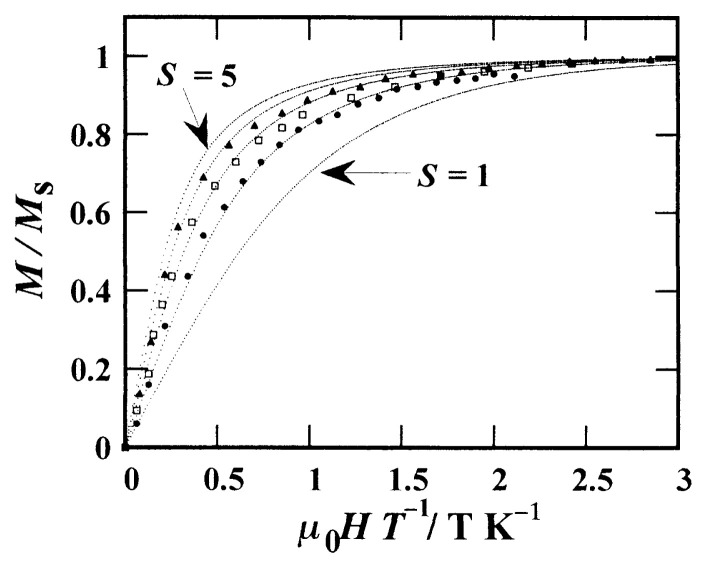
Magnetization of the 4:1, 6:1 and 9:1 mixed crystals of galvinoxyl and hydrogalvinoxyl at about 2 K plotted against ***μ***_0_*H/T*. The dotted curves correspond to the Brillouin function for *S* = 1, 2, 3, 4 and 5.

To comprehend the above matter in detail, the magnetic field dependence of the magnetization at 2 K was measured by making three types of the crystals of 4:1, 6:1, and 9:1 mixing ratios.11) The results are shown in Fig. 2. Depicted with the dotted curves are the theoretical curves of the Brillouin function for the spin quantum numbers *S* = 1, 2, 3, 4, and 5. When compared with the theoretical curves, it is seen that the experimental result for the crystal of *n*:1 mixing ratio almost corresponds to the theoretical curve for *S* = *n*/2. Therefore it turned out that *n* pieces of the galvinoxyl radicals of *S* = 1/2 are amassed as an average, and their magnetic moments are aligned in parallel at low temperatures. The crystal of galvinoxyl belongs to the monoclinic system (*C*2/*c*) and the almost planar radical molecules are arranged with the plane facing with each other along the *c*-axis at an equal interval.13) If it is assumed that the one-dimensional interaction along the *c*-axis is effective, the above results are easy to understand. That is, the one-dimensional chain of the radicals is divided into a number of segments comprised, on an average, of *n* radicals separated by the closed shell molecules having similar molecular structure, and the magnetic moments of the radicals are aligned by the ferromagnetic exchange interaction in parallel within the individual segments.11)

The magnitude of the exchange interaction was estimated to be 1.5 meV by using the electron paramagnetic resonance (EPR) absorption.14) The value corresponds well to the Weiss constant obtained from the susceptibility measurements.

## Mechanism of ferromagnetic interaction

### (a) SOMO-SOMO overlap

The mechanism governing the FM interaction is, of course, related to the electronic structure of the galvinoxyl radical and its crystal structure. We first examined the molecular orbitals (MO) of galvinoxyl by the INDO UHF method.

**Fig. 3:**
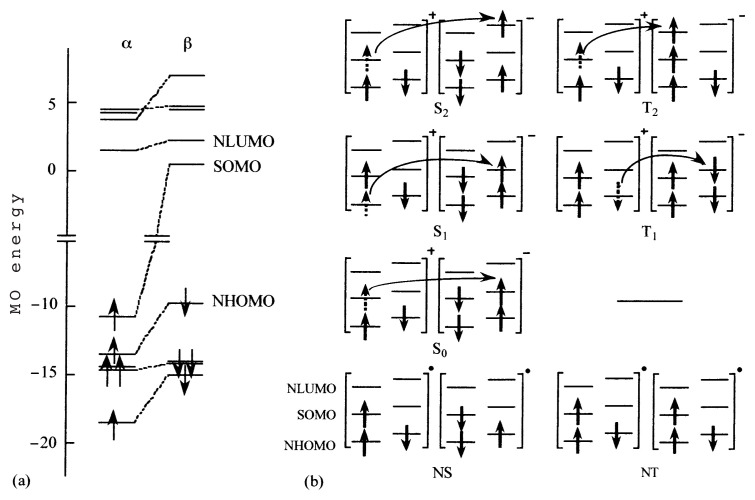
(a) ***π***-MO energy levels of galvinoxyl as calculated by UHF method. Only the levels near SOMO are presented. (b) The electronic configurations in a radical pair coupled by CT interaction.

The ***π***-MO energy levels of galvinoxyl near the singly occupied molecular orbital (SOMO) level are depicted in Fig. 3(a),15)–17) where the ***α***-spin orbitals and the corresponding ***β*** -spin orbitals are connected with dotted lines. The most conspicuous feature of this figure is that there exists the next highest occupied MO level of ***β*** -spin (NHOMO-***β***) situated higher than the SOMO of ***α***-spin (SOMO-***α***). The exchange interaction within the molecule is great enough to stabilize the SOMO-***α***. In other words, the spin correlation causes a large spin polarization effect in galvinoxyl.

As was mentioned above, the AFM interaction most frequently encountered among a number of organic radicals is explained by means of the analogy with the hydrogen molecules. Two neighboring radicals have a tendency to form a weak covalent bond by making a pair of the unpaired electrons. For the formation of a covalent bond, overlap of the orbitals of the unpaired electrons plays an important role. With galvinoxyl where directions of the spins are prone to be in parallel, how is the overlap of the orbitals? This point was examined.

The intermolecular overlap integral was calculated with SOMO’s using the molecular location of the crystal structure. The value of the SOMO-SOMO overlap became very small. When the overlap was calculated by changing the relative location of the molecules step by step, it turned out that the overlap at the actual molecular location in the crystal is close to the minimum.

Furthermore when the calculation process was carefully examined, it was found that relatively large positive and negative contributions cancel out each other, resulting in the small value. Since the ***π***-orbitals are spread out over the molecule, the partial overlaps become positive and negative here and there, and cancel out each other. As a result, the SOMO’s on the adjacent radicals are nearly orthogonal and degenerate. This situation is similar to the case of the d-orbitals of an isolated transition metal ion. In the case of d-orbitals on one center, the partial overlap becomes positive and negative, but cancels out to null and the d-orbitals are orthogonal and degenerate. The exchange integral becomes positive in such a case and the high spin state is stabilized in accordance with the Hund rule. With galvinoxyl, even though it is a multicenter molecule and the intermolecular interaction is now considered, the Hund rule may be applied in a modified manner and the spin state must be stable when the spins are maintained in parallel. This is the so-called potential exchange.

### (b) Charge-transfer interaction

The average distance between the neighboring galvinoxyl radicals along the *c*-axis is as much as 4.05 Å, and the positive and negative overlaps themselves are not very large. Accordingly it appears that the interaction large enough to be 1.5 meV cannot be explained only by the potential exchange. Some other mechanism, namely charge-transfer (CT) interaction, should also be taken into account.

In Fig. 3(b), some low energy CT configurations are shown for the neighboring pair of radicals.15)–17) NS and NT are the no-bond structures of singlet and triplet multiplicity, and S_i_ and T_i_ are the excited CT configurations, respectively. Among the excited CT configurations, S_0_ is the lowest and the resonance between S_0_ and NS usually stabilizes NS, resulting in an AFM interaction. This is the reason why most organic radicals exhibit AFM intermolecular coupling. With galvinoxyl, however, the SOMO-SOMO overlap is very small as mentioned above. Therefore, the stabilization of NS by admixture of S_0_ is to be minimized. On the other hand, T_1_ and T_2_ must be lower in energy than S_1_ and S_2_, respectively. In particular, T_1_ and T_2_ are much stabilized with respect to S_1_ and S_2_ in galvinoxyl because of the large spin polarization effect. Thus stabilization of NT by admixture of T_1_ and T_2_ is expected to overweigh that of NS, and the ground state becomes magnetic. It is in this way that the FM interaction is brought about in galvinoxyl.

For the sake of assurance, the intermolecular overlap integrals, which are thought to be proportional to transfer integral, are calculated for the orbitals relevant to the above configurations with the molecular arrangement in the crystal. The results are given in Table I.15)–17) As is seen, the overlap integrals for the T_1_ and T_2_ configurations are, respectively, larger than those for S_1_ and S_2_. Thus it is concluded that the off-diagonal interaction is also favorable for the NT stabilization.

**Fig. 4:**
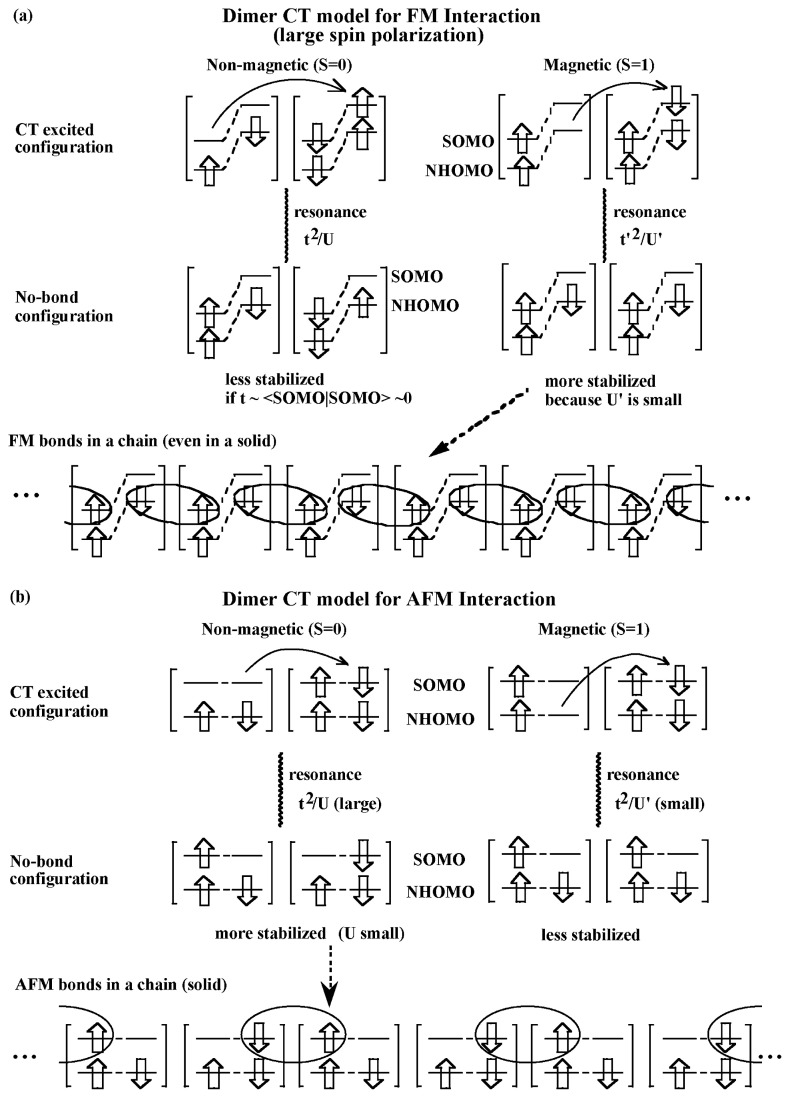
Schematic illustration of (a) ferromagnetic and (b) antiferromagnetic intermolecular bond formation. The bonds are shown by the elliptic circles.

From these observations, it is concluded that *the cooperative effect of the spin polarization caused by a large exchange interaction within a molecule and the virtual charge-transfer interaction among molecules is essential for the FM interaction* in the galvinoxyl crystal and probably for that in organic radical crystals in general.15) The arguments may be summarized schematically as those shown in Fig. 4, where the states, S_2_ and T_2_, are omitted for simplicity. In the case of FM coupling, the electrons in SOMO and NHOMO play an important role to make an intermolecular bond (Fig. 4a) because the overlap between them is large enough. The case of AFM interaction is shown in Fig. 4(b), where the overlap between SOMO’s is important for weak intermolecular bond formation.

From these, the requirements of the FM intermolecular interaction are summarized as follows: (1) large spin polarization within a molecule, and (2) small SOMO-SOMO overlap and large SOMO-NHOMO overlap between neighboring radicals.

The requirement (1) states the condition a radical molecule has to fulfil. The concept of spin polarization was well studied in the 1960’s, particularly in odd-alternate organic compounds, such as galvinoxyl and nitronyl nitroxide radicals. The spin polarization originates from an exchange interaction in a radical molecule. On the other hand, the requirement (2) is related to intermolecular interactions and to relative location of the neighboring radicals in a crystal. According to these, the FM intermolecular interaction originates in the exchange interaction in a molecule, which is always ferromagnetic. If the latter interaction is strong enough, it spreads out over a crystal through intermolecular CT interaction.

## Conditions for FM interaction in organic crystals

In the preceding section, we have examined the FM interaction in the galvinoxyl crystal. Now we extract, from the study on galvinoxyl, conditions for FM interaction applicable to other organic radicals.

As the property of radicals, it is first of all necessary for the spin polarization to be large enough. In other words, the exchange interaction within a molecule should be large. To obtain large exchange interactions in a molecule consisting of a number of atoms, it is recommended to use radicals having hetero-atoms such as nitrogen, oxygen and so on.15) Since these hetero-atoms have large electro-negativity, the unpaired electron in a ***π***-orbital has a large occupation possibility on the heteroatoms and interacts with the non-bonding electrons on these atoms. This interaction is expected to be very large because of its one-center character, and it almost determines the magnitude of the exchange interaction of the molecule itself, even though the unpaired electron is distributed over the molecule. In galvinoxyl, the oxygen atoms at the both terminals play this role.

If the exchange interaction within a molecule is very large, the CT configuration T_1_ of Fig. 3(b) is much stabilized and the energy of T_1_ comes very close to that of S_0_. This means that the exchange interaction almost overwhelms the energy interval between SOMO and NHOMO; namely SOMO and NHOMO behave as if they were degenerate. In order for this to be realized, a molecule having an extended ***π***-system is advantageous, because the interval between SOMO and NHOMO relates with the size of the ***π***-system.15) The extension of this argument to a radical having large exchange and extended ***π***-system will lead us to the situation where T_1_ comes below S_0_ energetically; this situation is similar to that proposed by McConnell for FM coupling in 1967.2)

The next requirement is a problem of crystal structure. As mentioned above, the SOMO-SOMO overlap should be minimized. This is a problem how to locate the radicals in a crystal. Although it is not easy to control the crystal structure, some trial has been applied by changing the position or size of chemical substituents and by introducing a charge or hydrogen bonding. However, the condition in favor for ferromagnetism was hereby pursued from the viewpoint of the electronic property of radicals.

**Fig. 5:**
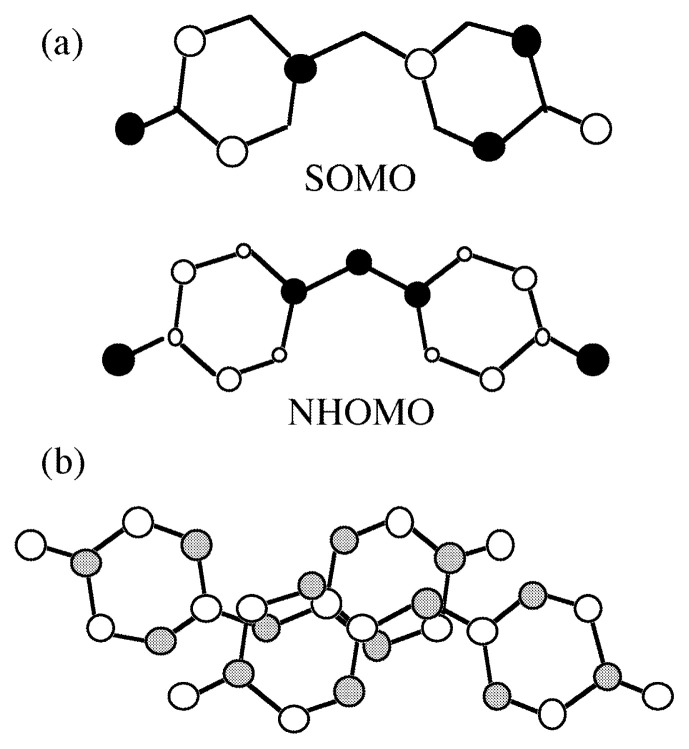
(a) Schematic drawing of SOMO and NHOMO of galvinoxyl (the *t*-butyl groups are omitted). Filled and open circles show the difference in polarity of the p-orbitals. (b) The molecular location of adjacent radicals in the crystal viewed along the normal to the molecular plane. SOMO has nodes at the atoms with shaded circles.

The galvinoxyl radical is long known as a radical with large spin polarization in the field of chemistry and has been studied as an example which possesses the so-called negative spin density. The negative spin density is usually observed with a radical of an odd-alternate ***π***-system. The feature of an odd-alternate system lies in SOMO; SOMO has nodes alternately on the bonded atoms and lobes with opposite polarities on both the neighboring atoms. Therefore, when two planar odd-alternate radicals come close to each other in a face-to-face manner and are relatively deviated by one bond or odd number of bonds, the local overlap is expected to become small because the node and the lobe overlap each other. In Fig. 5, a schematic diagram of SOMO of galvinoxyl and the relative location of the neighboring radicals are illustrated. It is easily seen that the local overlap is well effaced with each other, resulting in a minimal SOMO-SOMO overlap through the whole of the molecules. Thus there is a high possibility to accomplish such relative location of small SOMO-SOMO overlap by the use of the radicals of the odd-alternate system.15)

This situation may be looked from another point of view. Even though the charge density is null, a finite negative spin density is induced at the nodes by the electronic correlation (or exchange interaction) effect. There are positive spin densities on the other atoms locating at the lobes. Therefore, the relative face-to-face location of two radicals deviating by one or odd number of bonds is corresponding to the situation where the atom of negative spin density in one molecule comes on the atom of positive spin density in the other molecule. This situation is similar to the proposal for FM interaction by McConnell in 1963.1) From these, it seems that the two McConnell’s proposals are not independent of each other.

Another advantage of the use of the odd-alternate system is as follows: There is a high probability of the overlap of SOMO with NHOMO or NLUMO being enhanced once such relative location is established,15) because the charge density is usually distributed all over the molecules in the ***π***-MO’s other than SOMO.

The arguments given in this section are summarized as follows: The radicals of odd-alternate system having hetero-atoms and relatively developed ***π***-conjugation provide a high possibility of realizing the intermolecular FM interaction in a crystal.15)

## *p*-Nitrophenyl nitronyl nitroxide (*p*-NPNN)

### (a) Electronic and crystal structure of *p*-NPNN

**Fig. 6:**
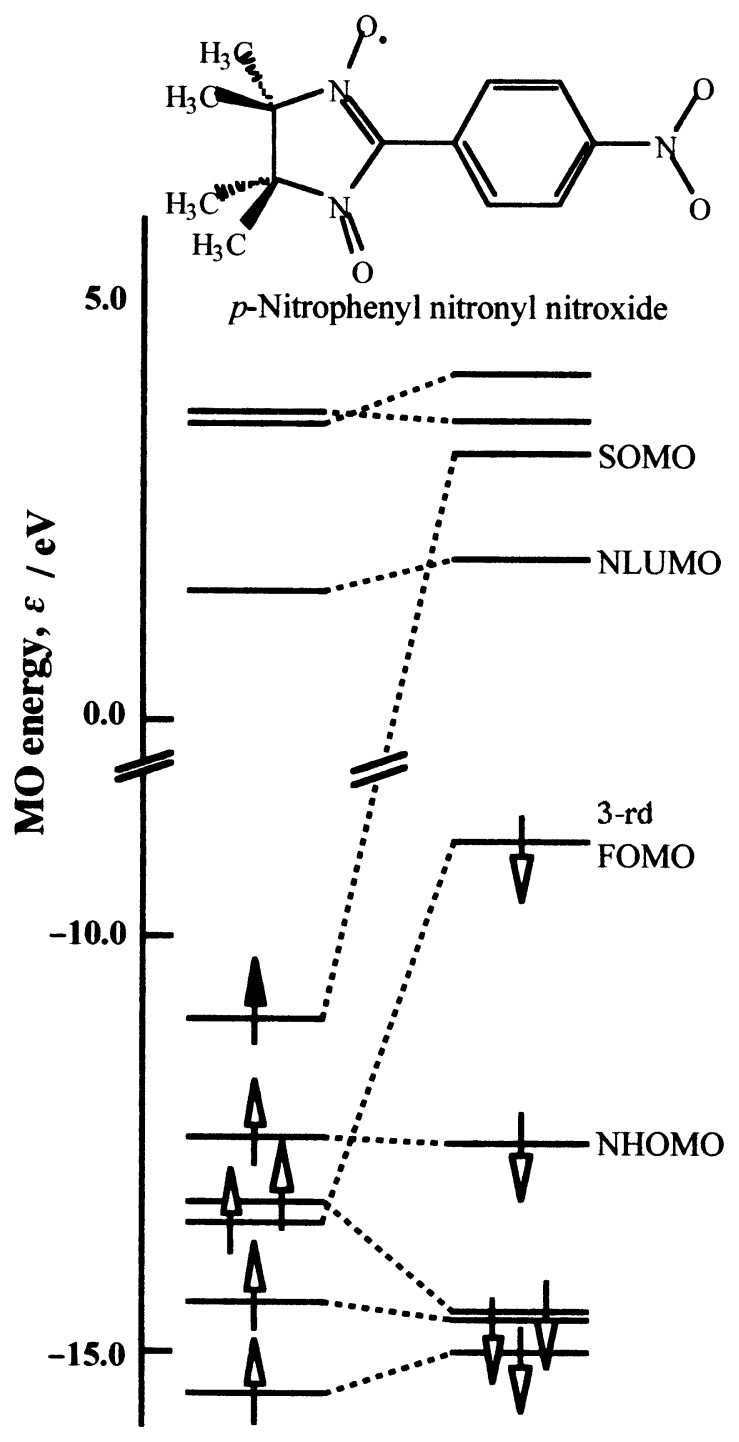
***π***-MO energy levels of *p*-NPNN. Only the levels close to SOMO are shown. FOMO means a fully occupied molecular orbital.

The first radical employed fulfilling the conditions in the preceding section is *p*-nitrophenyl nitronyl nitroxide (*p*-NPNN, see Fig. 6; IUPAC nomenclature, 2-(4-nitrophenyl)-4,4,5,5-tetramethyl-4,5-dihydro-1*H*-imidazol-1-oxyl-3*N*-oxide). Its large spin polarization effect has long been an object of research in the field of chemistry. When the molecular orbitals are actually calculated, the spin polarization effect manifests itself in a more obvious manner than in galvinoxyl; *i.e.*, the third fully occupied MO (FOMO) for the ***β*** -spin maintains higher energy than SOMO-***α*** as shown in Fig. 6.

**Fig. 7:**
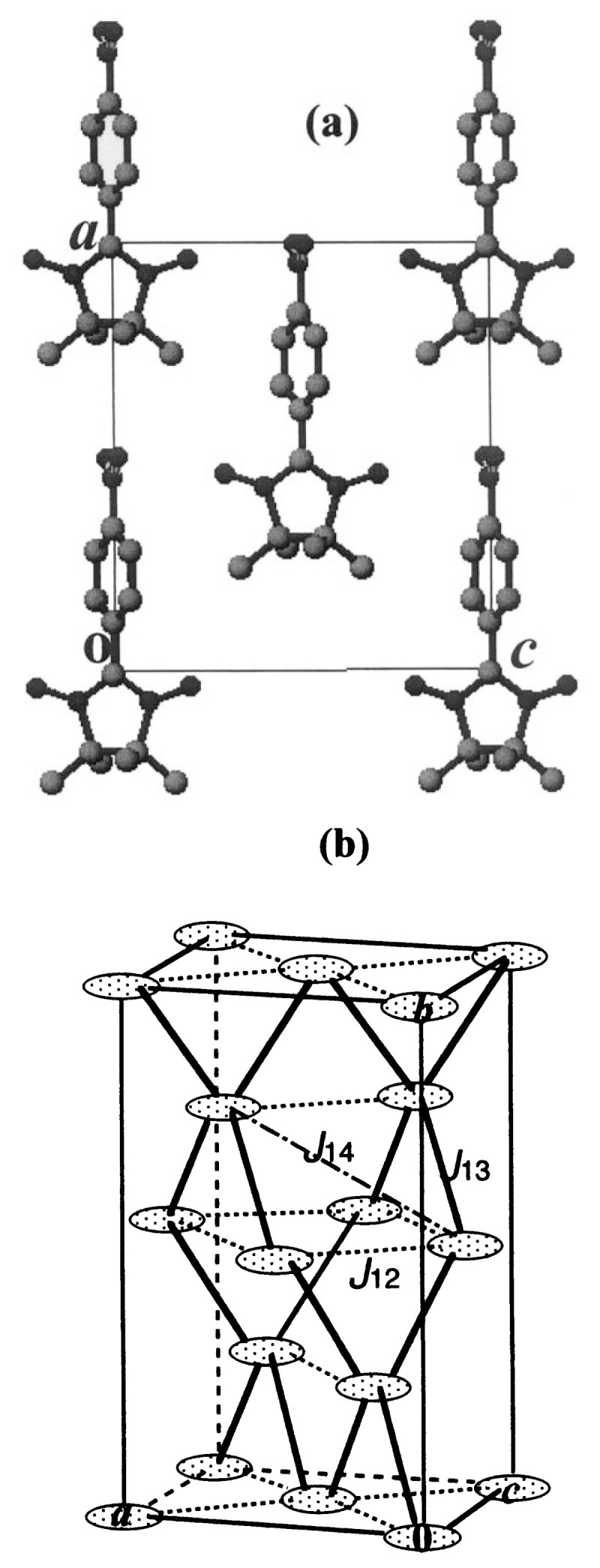
(a) The molecular arrangement on the *ac*-plane in the ***β*** - phase crystal. (b) Schematic illustration of the crystal structure. Each ellipsoid represents the *p*-NPNN radical molecule.

Also, the charge density in SOMO is mostly concentrated on the two NO moieties of the five-membered ring and only a little is distributed on the other parts of the molecule. Accordingly, it is expected that the SOMO-SOMO overlap minimizes provided that the NO moieties of the neighboring radicals do not approach each other in the crystal (this actually holds in the ***β*** - phase crystal, see Fig. 7). On the other hand, other frontier orbitals have the charge distribution ranging over the whole molecule, and the overlap with SOMO becomes large. These are favorable for FM interaction, as discussed above.

There are four polymorphic forms, ***α***-, ***β*** -, *γ* -, and ***δ*** - phase, known in *p*-NPNN.18)–21) Each of them can be separately prepared by properly adjusting the conditions for depositing crystals from solutions.22),23) The crystallographic constants of these phases are given in Table II. The orthorhombic ***β*** -phase is the most stable form, and the other forms are subject to change to the ***β*** -phase when they are kept at room temperature or below room temperature. The relation among these phases is recently examined in detail.23)

The molecular arrangement on the *ac*-plane of the ***β*** -phase crystal is shown in Fig. 7(a). The molecules are arranged in a parallel manner with the long molecular axis along the *a*-axis. Since the crystal belongs to the *F*2*dd* space group, the lattice can be divided into two face-centered orthorhombic sublattices, each deviating by *a*/4, *b*/4 and *c*/4. Thus the crystal structure is similar to that of diamond or, more precisely, zinc-blende, as shown schematically in Fig. 7(b), where the radical is denoted by an ellipsoid for simplicity. All the molecules on the *ac*-plane at *y* = 0 are tilted in one way and those at *y* = *b*/4 are in the other way with respect to the *ac-*plane. The best fit planes of the ONCNO moieties are tilted by ± 18.40 º from the *ac*-plane, those of the phenyl rings by ± 68.45 º and those of the nitro groups by ± 84.70 º.

### (b) Ferromagnetic interaction in the crystal

**Fig. 8:**
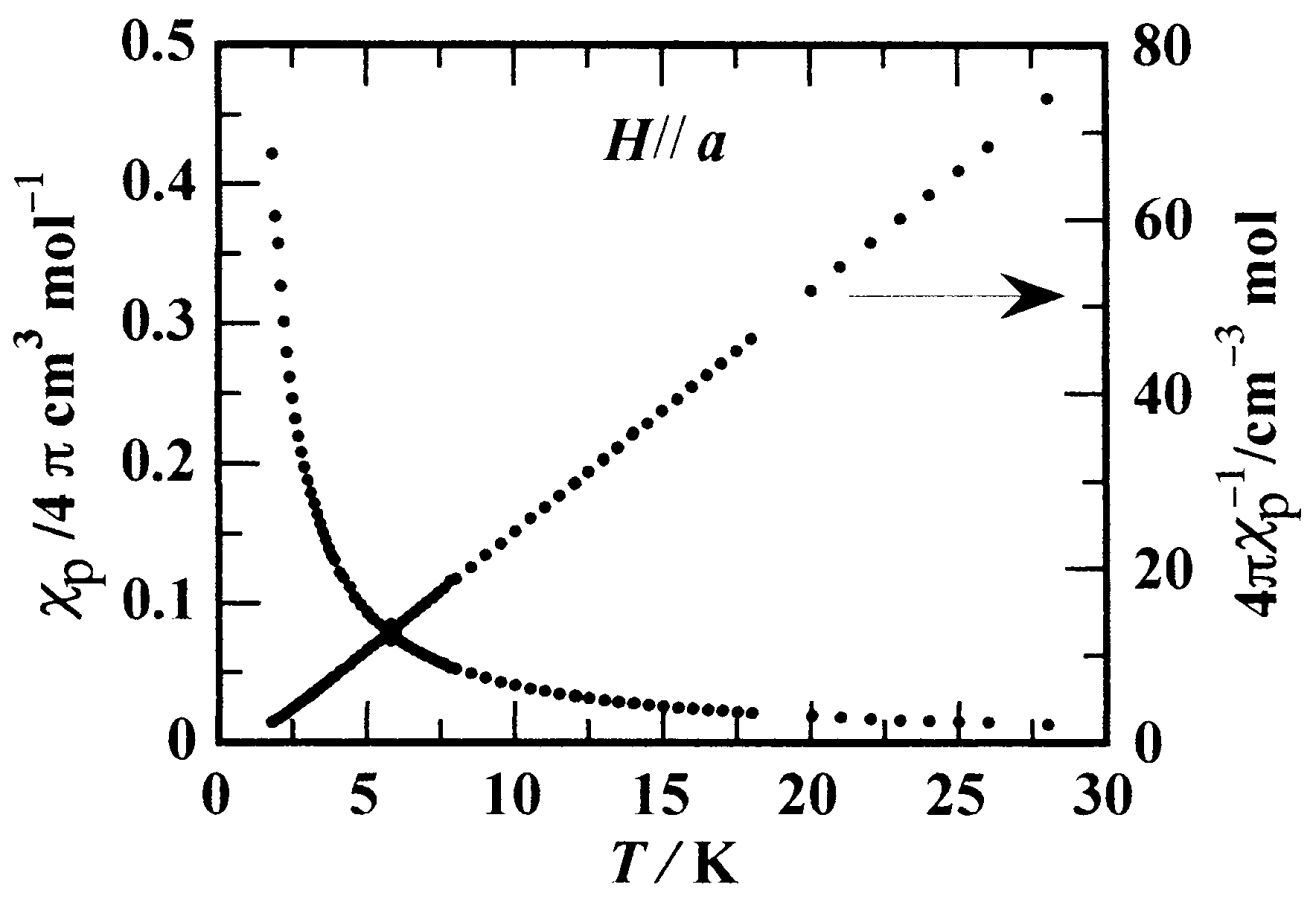
Temperature dependence of paramagnetic susceptibility of the ***β*** -phase of *p*-NPNN.

The magnetic property of the ***β*** -phase crystal of *p*-NPNN was first reported in 1989 by Awaga and Maruyama.24) The temperature dependence of the paramagnetic susceptibility follows the Curie-Weiss law and gives the ferromagnetic Weiss constant, ***Θ***, of about 1 K. In Fig. 8, our result of measurements with *H*//*a* is shown.8),22),25) There is no sizable anisotropy in the susceptibility, and nearly the same results are obtained, within the range of errors, in the other directions of the applied field.

**Fig. 9:**
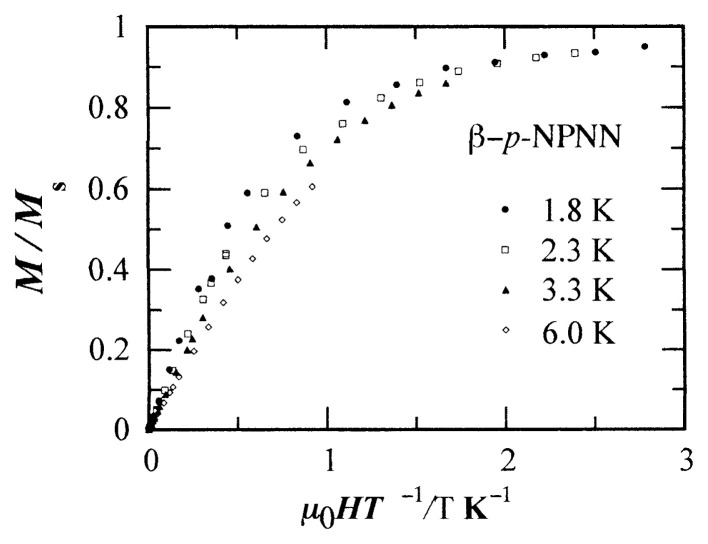
Magnetization of ***β*** -phase *p*-NPNN plotted against ***μ***_0_*H/T* at low temperatures. FM impurity.

Since the Weiss constant, ***Θ***, is very small, the FM interaction is also to be checked by measuring the field dependence of the magnetization at low temperatures. 8),22) Figure 9 shows the results. The lower the temperature is, the steeper the rise of the magnetization curve is. This indicates that the spins are connected by means of FM interaction.

The magnetization curves at several temperatures are unified into a single curve of the Brillouin function for *S* = 1/2 by the molecular field correction25) (not shown). In this case, the best fit is obtained with the coefficient of *λ* = 2.8 Oe mol emu^−1^ in *H*_eff_ = (*H* + *λ M*), which yields an FM Weiss constant of ***Θ*** = 1.1 K in agreement with the result of susceptibility measurements. In addition, this experiment assures that the sample is free from an FM impurity.

### (c) Transition to a ferromagnetic-ordered state

**Fig. 10:**
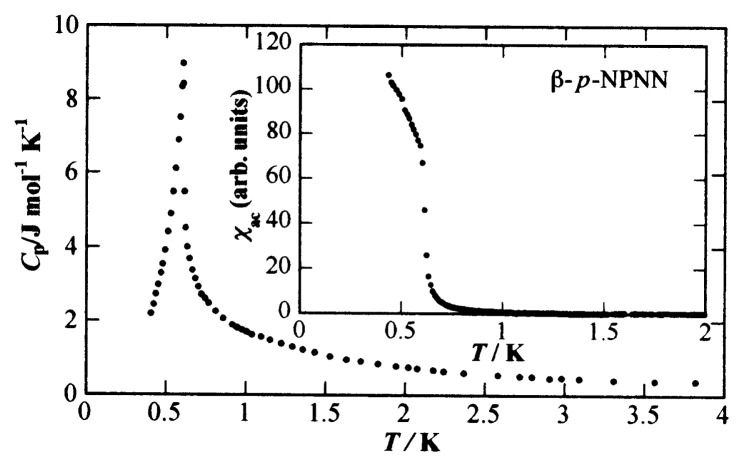
Main frame shows temperature dependence of the heat capacity and the inset shows ac susceptibility of ***β*** -phase *p*-NPNN.

In Fig. 10, the temperature dependence of the heat capacity and the ac susceptibility is shown.8),22),26) The heat capacity has a sharp peak at *T*_C_ = 0.6 K, and reveals the existence of a transition. The corresponding entropy amounts to 85% of *R*ln2 in the range up to 2 K. Thus it is concluded that the transition is magnetic and bulk in nature. As the ac susceptibility diverges around *T*_C_, the ordered state is, without doubt, a ferromagnetic state.

The magnetization curves measured at temperatures above and below *T*_C_ = 0.6 K are illustrated in Fig. 11.8),22),26) Although the curves have slight gradients at 1.22 and 0.81 K in the paramagnetic region, the data at 0.44 K clearly trace a hysteresis loop characteristic of ferromagnetism. The magnetization is almost saturated at about 50 Oe (1 Oe = 10^3^/4***π*** A m^−1^), and the coercive force is small. The reason for small coercive force is due to the facts of small anisotropy in *g*-factor and in dipolar interaction. The *g*-factors observed in the paramagnetic resonance are *g*_a_ = 2.0070, *g*_b_ = 2.0030, and *g*_c_ = 2.0106.22) The EPR linewidth is almost independent of field direction.

### (d) Other evidence for ferromagnetism

**Fig. 12:**
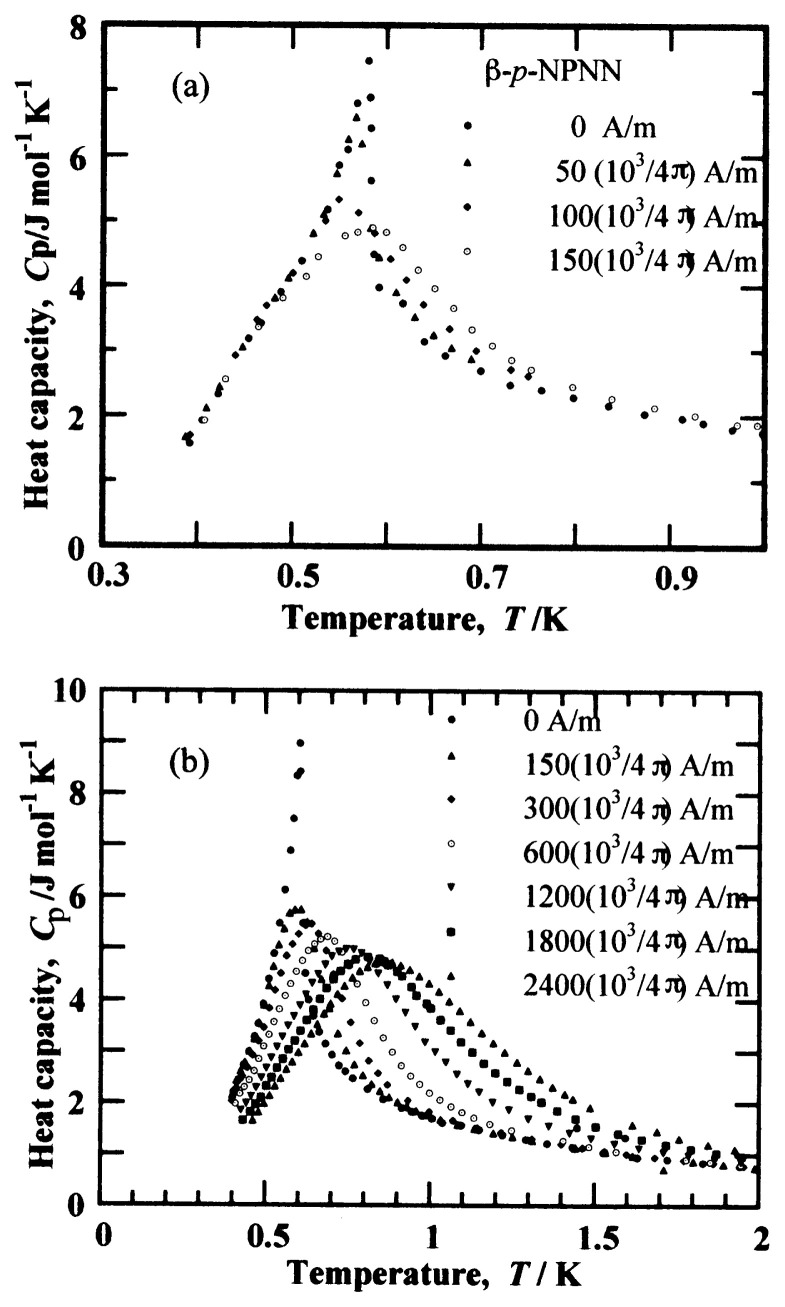
Temperature dependence of the heat capacity of ***β*** - phase *p*-NPNN at various applied magnetic field strengths. (a) Low and (b) high field region.

Illustrated in Fig. 12 is the temperature dependence of the heat capacity at various magnetic field strengths.22) As the magnetic field increases, the sharp peak in the zero field is slightly rounded in due course, and shifts to the higher temperature side. This is a feature of the ferromagnetic materials. For FM substances, the critical temperature cannot be defined in a finite magnetic field. When there is a FM interaction among the spins, they have a tendency to align themselves in parallel along the magnetic field at low temperature and the spin system becomes ordered by a weak field even slightly above the FM transition temperature. Thus the sharp peak of the heat capacity shifts and becomes rounded. In the case of antiferromagnetic order, the peak remains up to a certain field strength. Therefore, this experiment ensures the ferromagnetism of the ***β*** -phase crystal below 0.6 K.

**Fig. 13:**
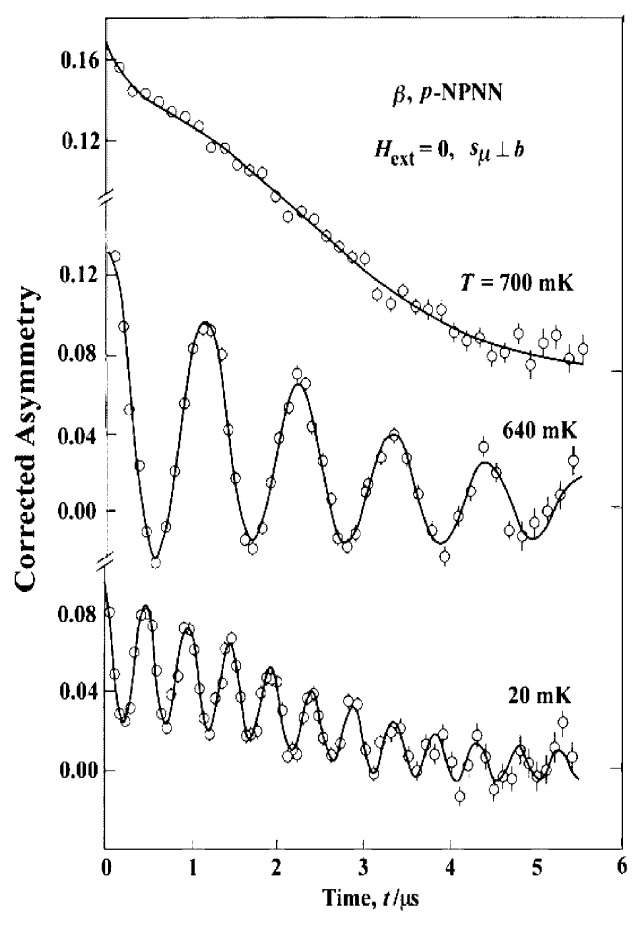
ZF-***μ*** SR time spectra observed on the ***β*** -phase single crystals of *p*-NPNN with initial muon spin polarized perpendicularly to the *b*-axis.

Another evidence for the ferromagnetism was obtained by the measurements of the zero-field muon spin rotation (ZF-***μ*** SR). Figure 13 shows some of the results of ZF-***μ*** SR experiments performed with the initial spin polarization perpendicular to the *b*-axis.27),28) The oscillating signals observed at 640 and 20 mK are due to the precession of the muons implanted into the crystal. Since there is no applied field, it is obvious that the internal field from the spontaneous magnetization causes the precession. The long-lasting oscillation indicates that the muons experience a rather homogeneous local field, which requires that the FM spin network is commensurate with the crystallographic structure.

Figure 13 shows the result for the muon spin polarization perpendicular to the *b*-axis. The amplitude of the oscillating signal diminishes to about 20% when the initial muon spin polarization is parallel to the *b-*axis. 27) This suggests that the spin orientation in different domains is not aligned randomly and is most likely along the *b*-axis. Recent FM resonance experiments by Ohshima29) and neutron diffraction measurements by Schweizer’s group30),31) also show the magnetic easy axis is along the *b*-axis in the ***β*** -phase. The magnetic dipole interaction, ***D***, in the ordered state was calculated using the spin density data obtained by the neutron diffraction experiment.30) The results are *D*_a_/*k*_B_ = − 0.016, *D*_b_/*k*_B_ = − 0.029 and *D*_c_/*k*_B_ = 0.045 K.33) This also indicates that the spin system is most stable when the spins are aligned in parallel along the *b*-axis.

**Fig. 14:**
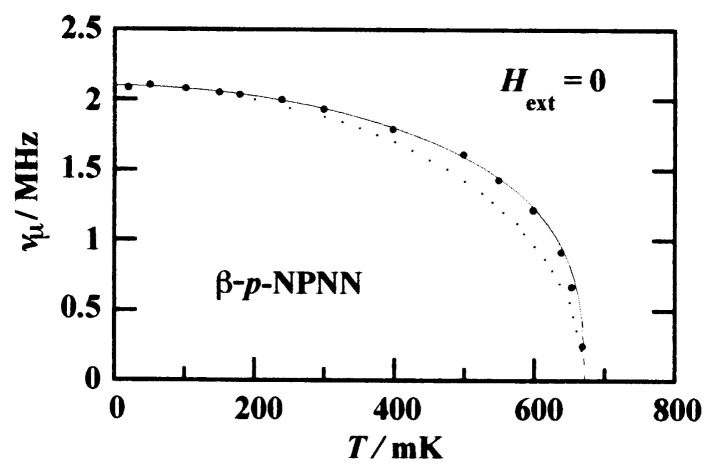
Temperature dependence of muon spin precession frequency in ***β*** -phase crystals below *T*_C_ in zero external field. The frequency is proportional to the spontaneous magnetization.

The oscillation frequency is approximately related to the internal field by ***ν****_μ_* = (*γ_μ_*/2***π***)*B*_int_, where the muon gyromagnetic ratio *γ_μ_*/2***π*** = 135.53 MHz T^−1^. In Fig. 14, the frequency is plotted against temperature.27) The frequency extrapolated to 0 K corresponds approximately to the local field of 15.5 mT.32) The solid line in Fig 14 shows a fit of *M*(*T*) ∝ *M*(0)[1 − (*T/T*_C_)*^α^*]*^β^* with ***α*** = 1.86 and ***β*** = 0.32 (***α*** = 1.74 and ***β*** = 0.36 are reported by Blundell *et al.*34)). The agreement between the ***μ*** SR results and the solid line is good. This allows us to discuss the results in two interesting regions, namely *T* → 0 K and *T* → *T*_C_. At temperatures well below *T*_C_, *M* decreases with increasing temperature as [*M*(0) − *M*(T)] ∝ *T^α^*, close to the magnon-like behavior of [*M*(0) − *M*(T)] ∝ *T*^1.5^. Near *T*_C_, *M*(*T*) ∝ (*T*_C_ − *T*)*^β^* with the critical magnetization exponent ***β*** = 0.32, in agreement with the value of 1/3 expected for three-dimensional Heisenberg system. The temperature dependence of *M*(*T*) in ***β*** - phase *p*-NPNN is thus consistent with that of three-dimensional Heisenberg system both at low temperature and near *T*_C_.

The dotted curve shown is the one calculated using the random phase approximation on the assumption of a three-dimensional isotropic Heisenberg model with a specific choice of 2*J/k*_B_ = 470 mK for the interactions with the eight neighboring radicals. These results of the ZF-***μ*** SR experiments clearly demonstrate the appearance of spontaneous magnetic order in the ***β*** - phase crystal of *p*-NPNN.

Although the 2*J/k*_B_ value of 470 mK is cited above only as an average, the exchange interaction is supposed not to be only one kind. From the crystal structure, schematically shown in Fig. 7(b), we should consider at least three kinds of near neighbor interactions, *J*_12_, *J*_13_, and *J*_14_ as indicated. According to the calculation by Okumura *et al.*, the interaction is estimated to be 2*J*_12_/*k*_B_ = 0.48 K., 2*J*_13_/*k*_B_ = 0.22 K and *J*_14_ to be slightly antiferromagnetic.35)

### (e) Pressure effect

The ferromagnetism of the ***β*** - phase crystal of *p*-NPNN was established by the experiments described above. The pressure effect on the magnetic properties was then examined up to 10.4 GPa.36)–38) Here, some results of the pressure dependence of ac susceptibility are briefly described.

The critical temperature as a function of pressure, *T*_C_(*p*), shifts towards the lower temperature side with the initial gradient d[*T*_C_(*p*)/*T*_C_(*p*_0_)]/d*p* = − 0.48 GPa^−1^, and the magnitude of the susceptibility decreases gradually as the pressure increases from *p*_0_ (= 0 MPa). In the low-pressure region below about 650 MPa, however, the ferromagnetic behavior is preserved below *T*_C_(*p*). In the high-pressure region above about 650 MPa, on the contrary, the magnitude of the susceptibility becomes quite small, the susceptibility in the ordered state decreases sensitively with the pressure increase, and the shoulder-like curve of *χ*_ac_ around *T*_C_(*p*) changes into a cusp. Furthermore, *T*_C_(*p*) turns to increase as the pressure increases with a gradient of d[*T*_C_(*p*)/*T*_C_(*p*_c_)]/d*p* = + 0.04 GPa^−1^, where *p*_c_ = 650 ± 50 MPa is the critical pressure. These results suggest that the magnetic order below *T*_C_(*p*) is of an antiferromagnet under high pressure. Therefore, it was concluded that the ***β*** -phase crystal undergoes a pressure-induced transition from the ferromagnetic to the antiferromagnetic phase at about 650 MPa.

## Other organic ferromagnets

Following the finding of the first example of an organic ferromagnet in the ***β*** -phase *p*-NPNN, a dozen organic compounds have been found to become a ferromagnet.39)–46) The highest *T*_C_ is 1.48 K for diazaadamantane dinitroxide.41) Their ferromagnetism are mostly characterized by the measurements of ac susceptibility and magnetization, and some by the heat capacity measurements. In some cases, however, more detailed examination is required in order to establish their ferromagnetism.

## Figures and Tables

**Fig. 11:**
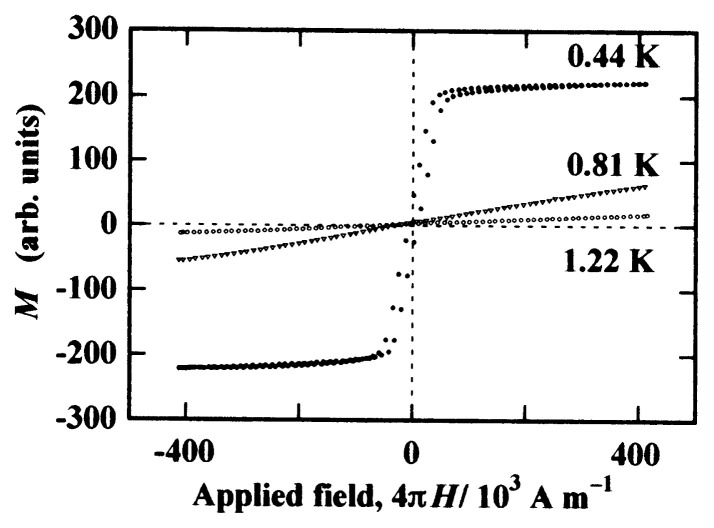
Magnetization curves of ***β*** -phase *p*-NPNN above and below *T*_C_ = 0.6 K.

**Table I:** Calculated intermolecular overlap integrals for the CT configurations in Fig. 3.

S_0_	<SOMO-***α***|SOMO-***β***>	0.72 × 10^−3^
T_1_	<SOMO-***β*** |NHOMO-***β***>	1.60 × 10^−3^
S_1_	<SOMO-***β*** |NHOMO-***α***>	0.87 × 10^−3^
T_2_	<SOMO-***α***|NLUMO-***α***>	2.73 × 10^−3^
S_2_	<SOMO-***α***|NLUMO-***β***>	1.33 × 10^−3^

**Table II:** Crystallographic constants of the four phases of *p*-NPNN

Phase	***α***-phase	***β*** -phase	*γ*-phase	***δ***-phase
System	monoclinic	orthorhombic	triclinic	monoclinic
Space group	*P*2_1_/*c*	*F*2*dd*	*P*1̄	*P*2_1_/*c*
*a*/Å	7.307	12.347	9.193	8.963
*b*/Å	7.596	19.350	12.105	23.804
*c*/Å	24.794	10.960	6.471	6.728
***α***/deg			97.35	
***β***/deg	93.543		104.44	104.25
*γ*/deg			82.22	
*Z*	4	8	2	4
*V*/Å^3^	1373.5	2618.5	687.6	1391.3
